# The prevalence of arterial hypertension in Morocco: Screening results from ‘May Measurement Month’ 2024

**DOI:** 10.21542/gcsp.2025.51

**Published:** 2025-10-31

**Authors:** Nora Taiek, Abderrahmane Belkacem, Faiza Aziouaz, Abdelkader Jalil El Hangouche

**Affiliations:** Department of Physiology, Faculty of Medicine and Pharmacy of Tangier, Abdelmalek Essaâdi University

## Abstract

Background: Arterial hypertension is the leading cause of mortality worldwide. The International Society of Hypertension initiated an annual global initiative called the May Measurement Month screening campaign to raise awareness regarding arterial hypertension and its adverse outcomes.

Aim: To evaluate the prevalence of arterial hypertension in Morocco, as well as the prevalence of uncontrolled and untreated hypertension.

Methods: Adult participants (≥18 years) were screened through opportunistic sampling across Morocco during May 2024. Trained volunteers recorded standardized blood pressure measurements, with the last two readings being averaged from community volunteer participants, along with socio-demographic parameters, weight, height, lifestyle factors, and comorbidities. Hypertension was defined as systolic blood pressure ≥ 140 mmHg and/or diastolic blood pressure ≥ 90 mmHg and/or taking antihypertensive medication.

Results: A total of 1421 participants were screened in May 2024, comprising 727 (51,2%) females and 694 (48,8%) males with a median age of 48 years (SD 20.1). Of all 1421 participants, 524 (36.9%) were hypertensive, while 52.9% were unaware of their hypertension status. Among participants who were previously diagnosed with hypertension, 79.4% were on antihypertensive medication, and only 45,9% had controlled hypertension.

Conclusion: Our study registered a high prevalence of arterial hypertension in Morocco. It also pointed to a high burden of undiagnosed and uncontrolled hypertension.

## Introduction

Over the past three decades, the global incidence of diseases has risen significantly^1^. The principal causes have notably shifted from communicable to non-communicable origins, including cardiovascular diseases (CVDs)^2^. This shift in disease patterns corresponds to the epidemiological transition attributed to socioeconomic progress and lifestyle changes^3^.

Annually, non-communicable diseases (NCDs) are responsible for 41 million deaths worldwide^4^, with more than three-quarters of cardiovascular disease-related deaths occurring in low- and middle-income countries^2^. In Morocco, 80% of total mortality is attributed to NCDs, of which 38% are cardiovascular diseases^1,5^.

Hypertension refers to abnormally high blood pressure values, defined as an office systolic blood pressure of 140 mmHg or higher or a diastolic blood pressure of 90 mmHg or higher^6^. Arterial hypertension represents a global public health challenge, being the primary risk factor for global mortality and the principal modifiable risk factor for cardiovascular diseases^7,8^. Often referred to as the “silent killer” due to its asymptomatic nature^9^, high blood pressure can remain undiagnosed for years, leading to severe complications such as heart disease, stroke, and chronic renal failure^8,10^.

Hypertension is linked to 10.4 million deaths annually^11^, contributing to 57 million disability-adjusted life years (DALY) globally^12^ and about 658,301 in Morocco^10^. The prevalence of arterial hypertension increased from 650 million to 1.3 billion between 1990 and 2019^13^.

Africa exhibits the highest prevalence globally, with 46% of the population over 25 years old affected^14^. Morocco is no exception, ranking among the countries with highest hypertension prevalence rates in the Eastern Mediterranean region^15^. In 2023, the World Health Organization released its first global report on arterial hypertension, which mentioned that only 54% of adults with hypertension are diagnosed, 42% receive treatment, and a mere 21% have controlled hypertension^9,16^. Therefore, these findings underscore the need for systematic blood pressure screening campaigns to raise awareness, ensure early diagnosis, and promote timely treatment and effective control of hypertension^9^.

Our study aims to assess the prevalence of arterial hypertension among the general population of Morocco based on the latest European Society of Cardiology Guidelines for the Management of Arterial Hypertension 2024. The study was conducted in the context of the international May Measurement Month (MMM) screening campaign, initiated by the International Society of Hypertension. This initiative was carried out for the first time in Morocco by the Physiology laboratory of the Faculty of Medicine and Pharmacy in Tangier as part of a larger research project called “The epidemiology of arterial hypertension in Morocco”.

## Methods

During the month of May 2024, a cross-sectional observational study was conducted among the Moroccan population. An estimated sample size of 385 was calculated using a margin of error of 5% and a confidence level of 95%. The inclusion criteria for study participants were designed to ensure that the mean age and gender distribution of our sample would be representative of the general population of Morocco, based on data from the Moroccan High Commission for Planning (HCP)^17^. Adolescents and children under the age of 18 years old were not included in our study, knowing that this category represents about 25.2% of the total Moroccan population according to HCP^17^.

A total of 1436 subjects aged 18 years and older, from various regions of Morocco voluntarily agreed to participate in our study; 15 participants were excluded due to missing informations about age, weight and height. Screenings took place at diverse public sites, including parks, train stations, shopping malls, and marketplaces.

All volunteers involved in data collection received initial training on standardized measurement procedures to ensure consistency and uniformity across various screening sites, and to guarantee the quality of the data collected, meanwhile supervisors took care of observing the field to assure adherence to the study protocol. Additionally, investigators were provided with an information leaflet detailing the study’s objectives and key facts about hypertension to enhance understanding and facilitate communication with participants.

Blood pressure was measured using a validated automated electronic blood pressure monitor (HEM9200T, OMRON Healthcare, Kyoto, Japan), following international guidelines for blood pressure monitoring^6^. Arm circumferences were measured to ensure the use of appropriately sized cuffs: a standard cuff for circumferences under 32 cm, a large cuff for those between 32 and 42 cm, and an extra-large cuff for circumferences exceeding 42 cm^6^.

For optimal measurement accuracy, participants’ blood pressure was preferably measured on the left arm. The cuff was placed at their heart level, with their backs supported, legs uncrossed, and resting flat on the ground. Participants were asked to abstain from smoking before and during the measurements and were informed to remain silent throughout the process^6^.

Initially, a preliminary BP measurement was performed, followed by a standardized five-minute rest period. During this interval, participants completed a standardized questionnaire designed to collect socio-demographic data, anthropometric characteristics, lifestyle factors, and health-related information. Subsequently, three BP measurements were recorded one minute apart. The final BP value considered for analysis was the average of the last two readings^6^.

Data were collected and transposed into an Excel spreadsheet then analyzed using the Statistical Package for the Social Sciences (IBM SPSS Statistics, IBM Corp., Version 26.0, Armonk, NY). Categorical variables were summarized as frequencies and percentages, while quantitative variables were described using appropriate summary statistics based on their distribution.

A Kolmogorov–Smirnov test was used to assess the distribution of our sample. Our data were not normally distributed; therefore, Mann–Whitney and Kruskal-Wallis tests were used for non-parametric comparisons of quantitative variables, whilst the chi-squared test was used for categorical variables. Statistical significance was set at a *p*-value threshold of < 0.05.

The study protocol received approval from the Ethics Committee of the University-Hospital of Tangier (AC68FV/2024) and was executed in compliance with the ethical principles of the Declaration of Helsinki (1964,amended 2023) and conformed to good clinical practice guidelines^18^.

For the assessment of arterial hypertension prevalence, the 2024 European Society of Cardiology (ESC) Guidelines for the Management of Arterial Hypertension were selected, as they incorporate the most current diagnostic thresholds and provide an updated classification of blood pressure into three categories; non-elevated (< 120/80 mmHg), elevated (120–139/80–89 mmHg), and hypertension (≥140/90 mmHg), based on the latest evidence and expert consensus.

While the hypertension threshold remains the same (140/90 mmHg) in both the 2024 ESC and the 2018 ESC Guidelines for Arterial Hypertension, the classification of blood pressure values differs. The 2018 ESC recommendations classify blood pressure values into optimal < 120/80 mmHg, normal (120 − 129/80 − 84 mmHg), high normal (130 − 139/85 − 89 mmHg), hypertension grade I (140 − 159/90 − 99 mmHg), hypertension grade II 160 − 179/100 − 109 mmHg, hypertension grade III (≥ 180/ 110mmHg), and isolated systolic hypertension ≥140/< 90 mmHg).

In this context, a secondary analysis was conducted by classifying blood pressure values according to the 2018 ESC Guidelines, with the aim of assessing the proportion of subjects classified as “Normal” based on the 2018 ESC guidelines who would be reclassified as “Elevated” under the revised 2024 ESC Guidelines for arterial hypertension. Knowing that subjects categorized as having elevated blood pressure should either follow lifestyle modification such as dietary changes, physical activity, reducing alcohol consumption, and smoking cessation, or initiate pharmacological treatment for patients with high cardiovascular risk^6^.

## Results

In May 2024, 1,436 subjects agreed to participate in our study, 15 were excluded due to missing information about age, weight and height. In total 1,421 Moroccans participated in the month-long screening campaign on arterial hypertension in Morocco. The median age of the participants was 48 years old (range: 18–90 years), with a balanced gender distribution, though females slightly predominated at 51.2%, which is representative to the general population of Morocco according to recent data of the High Commission for Planning (HCP) of Morocco^17^.

Anthropometric measurements showed that 19.6% of participants were obese, 38.8% were overweight, and less than 1% had morbid obesity. Regarding tobacco consumption, 14.2% were current smokers, and only 10.7% were former smokers. Furthermore, 19.3% of respondents reported a personal history of cardiovascular diseases (Table 1).

**Table 1:** Sociodemographic characteristics of study participants.

Characteristics	**n (%)**	**Median (Q1-Q3)**	**CIs 95%**
**Gender**			
*Male*	694 (48.8%)		46.3–51.5
*Female*	727 (51.2%)		48.5–53.7
**Age *(years)***		48 (35-61)	47–49
**Height *(cm)***		167 (160-174)	160.5–175
**Weight *(kg)***		72 (64-82)	65–83
**BMI *(kg/m*^2^)**		25.8 (23-29)	23.4–29.4
**BMI classification**			
*Underweight*	45 (3.2%)		2.3–4.2
*Normal*	545 (38.4%)		35.8–40.9
*Overweight*	552 (38.8%)		36.3–41.4
*Moderate Obesity (Class 1)*	219 (15.4%)		13.5–17.2
*Severe Obesity (Class 2)*	47 (3.3%)		2.5–4.2
*Morbid Obesity (Class 3)*	13 (0.9%)		0.4–1.5
**Educational level**			
*Illiterate*	258 (18.2%)		16.3–20.2
*Primary*	222 (15.6%)		13.7–17.5
*Secondary*	494 (34.8%)		32.3–37.2
*University*	447 (31.5%)		29–33.7
**Profession**			
*Student*	62 (4.4%)		3.3–5.4
*Unemployed*	442 (31.1%)		28.5–33.5
*Employee*	511 (36%)		33.5–38.4
*Liberal Profession*	249 (17.5%)		15.6–19.5
*Retired*	157 (11%)		9.5–12.8
**Marital status**			
*Single*	319 (22.4%)		20.3–24.7
*Married*	960 (67.6%)		65.2–70
*Divorced / Widow*	142 (10%)		8.4–11.5
**Smoking status**			
*Non-smoker*	1067 (75.1%)		72.7–77.2
*Smoker*	202 (14.2%)		12.4–16.1
*Ex-smoker*	152 (10.7%)		9.1–12.3
**Personal History of Cardiovascular Diseases**			
*Absence*	1267 (89.2%)		87.4–90.7
*Presence*	154 (10.8%)		9.3–12.6

Among the 1,421 participants, 36.9% were hypertensive based on the 2024 ESC Guidelines of arterial hypertension, and of these, only 47.1% were aware of their blood pressure condition. All hypertensive individuals were referred to their nearest primary health care facility, and all participants received an educational leaflet containing recommendations on dietary and lifestyle changes^19^. Out of 247 hypertensive participants, 79.4% were on anti-hypertensive medication. Though only 45.9% achieved blood pressure control^6^. Dietary and lifestyle modifications alone allowed 27 hypertensive participants to achieve controlled blood pressure without the use of medication. This contributed to an overall blood pressure control rate of 47.4% among all hypertensive individuals aware of their condition (Table 2).

**Table 2:** Total participants and proportions with hypertension awareness, on medication, and with controlled BP.

Characteristics	n (%)	CIs 95%
**Total participants: 1421**		
Number (%) with hypertension	524 (36.9%)	34.3–39.4
**Among Hypertensive Participants: 524**		
Number (%) of hypertensives aware	247 (47.1%)	42.6–51.5
Number (%) of hypertensives on medication	196 (79.4%)	74.1–84.2
Number (%) of those on medication with controlled BP	90 (45.9%)	47.4–61.2
Number (%) of those following lifestyle changes with controlled BP	27 (10.9%)	6.9–15
Number (%) of aware hypertensives with controlled BP	117 (47.4%)	40.5–53

Participants’ blood pressure classification was primally guided by the ESC 2024 guidelines; the classification of blood pressure as non-elevated, elevated, and hypertension was 10.6%, 52.5% and 36.9%, respectively. The classification according to the 2018 ESC guidelines showed that 30.3% of participants had optimal blood pressure, 17.7% had normal blood pressure, and 15.1% had high normal blood pressure. 33.9% of hypertensives had grade I hypertension, while only 5.9% had grade III. Of note, a quarter (25.9%) of hypertensives had isolated systolic hypertension, a condition particularly pertinent to aging population and related to higher CVD morbidity and mortality (Table 3).

**Table 3:** Blood pressure classification of study participants according to the European Society of Cardiology (ESC) hypertension guidelines of 2018 and 2024.

Blood pressure classification of Study participants	n (%)	SBP	DBP	CIs 95%
**According to the 2018 ESC guidelines**				
Optimal	430 (30.3%)	108.0	71.2	27.9–32.6
Normal	252 (17.7%)	121.3	78.6	15.8–19.8
High normal	215 (15.1%)	129.9	82.5	13.4–17
Grade 1 hypertension	178 (12.5%)	136.5	93.5	10.8–14.2
Grade 2 Hypertension	62 (4.4%)	154.2	100.6	3.4–5.4
Grade 3 Hypertension	31 (2.2%)	176.1	109.4	1.4–3
Isolated systolic hypertension	136 (9.6%)	149.8	80.5	8.0–11
Controlled hypertension	117 (8.2%)	122.6	76.7	6.9–9.7
**According to the 2024 ESC guidelines**				
Non- elevated	151 (10.6%)	104.745	64.656	9.1–12.2
Elevated	746 (52.5%)	119.452	78.279	49.9–55.3
Hypertension	524 (36.9%)	141.287	88.186	34.3–39.3

**Notes.**

SBP Systolic blood pressure DBP Diastolic blood pressure

**Table 4:** Results of logistic regression analysis of the association of hypertension status and sociodemographic status. *P* value significant at: Bold value: *p* < 0.001, Bold value* < 0.05.

Characteristics	**Hypertension status**		**Binary logistic regression**			
			**Univariate analysis**		**Multivariate analysis** **(** ***R*** ^**2**^ ***=0.257*** **)**	
	***Absent***	***Present***	***OR***	***95%***	***OR***	***95%***
**Gender**						
*Male*	437	257	1		1	
*Female*	460	267	0.987	0.796–1.224	0.789	0.583–1.068
**Age *(years)***						
*<40*	415	64	**1**		1	
*40–60*	306	225	**4.768**	3.481–6.530	**3.600**	2.527–5.128
*60–70*	129	129	**6.484**	4.529–9.284	**4.584**	2.960–7.098
*>70*	47	106	**14.624**	9.487–22.543	**10.061**	5.900–17.157
**BMI**						
*<25*	433	177	1		1	
*25–30*	323	213	**1.613**	1.261–2.063	**1.327**	1.003–1.754
*30<*	141	134	**2.325**	1.733–3.120	**1.976**	1.413–2.763
**Educational level**						
*Illiterate*	128	130	1		1	
*Primary*	132	90	0.671*	0.467–0.965	0.817	0.549–1.216
*Secondary*	326	168	**0.507**	0.373–0.690	0.808	0.569–1.148
*University*	311	136	**0.431**	0.314–0.591	0.823	0.555–1.219
**Profession**						
*Student*	59	3	1		1	
*Unemployed*	420	271	**12.690**	3.938–40.886	3.597*	1.041–12.431
*Employee*	351	160	**8.965**	2.769–29.029	3.869*	1.134–13.201
*Retired*	67	90	**26.418**	7.938–87.916	4.172*	1.140–15.275
**Marital status**						
*Single*	256	63	1		1	
*Married*	570	390	**2.780**	2.051–3.769	1.020	0.705–1.474
*Divorced / Widowed*	71	71	**4.063**	2.646–6.241	1.087	0.649–1.821
**Smoking status**						
*Non-smoker*	668	399	1		1	
*Smoker*	139	63	0.759	0.550–1.048	0.755	0.506–1.126
*Ex-smoker*	90	62	1.153	0.816–1.631	0.711	0.463–1.092
**Personal History of Cardiovascular Diseases**						
*Absence*	851	416	1		1	
*Presence*	46	108	**4.803**	3.336–6.915	**3.575**	2.407–5.308

**Notes.**

BMI Body Mass Index

These results showed that 17.7% of participants are classified as normal with the 2018 ESC guidelines would be reclassified as having elevated BP according to the 2024 ESC guidelines for arterial hypertension, and these individuals have to follow lifestyle modification such as healthy dietary habits, physical activity, reducing alcohol consumption, and smoking cessation, or start pharmacological treatment for patients with high cardiovascular risk.

**Figure 1.**
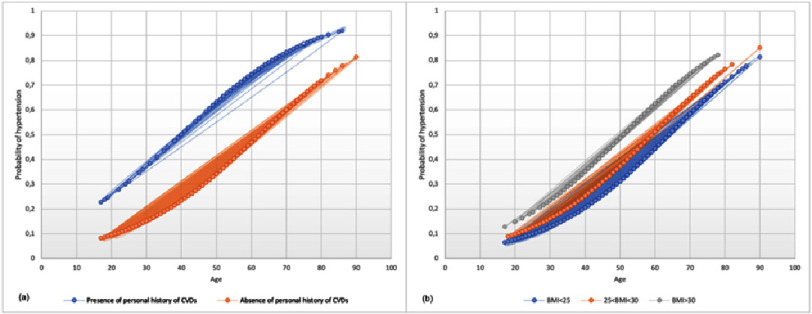
Predicted probability of developing arterial hypertension according to individual’s body mass index, BMI (a), and personal history of cardiovascular disease, CVDs (b).

All socio-demographic, anthropometric and lifestyle characteristics were significantly associated with hypertension, except, for gender (*p* = 0.905) and smoking status (*p* = 0.14) (Table 4). In multivariate logistic regression analysis, the predicted probability of an individual having hypertension remains significantly associated with older age, higher BMI and presence of personal history of cardiovascular diseases (Figure 1 a-b). Participants with a personal history of cardiovascular diseases have a higher predicted probability of developing hypertension than individuals without a personal history of cardiovascular diseases, and it increases with age (Figure 1 a). On the other hand, the probability of having hypertension was predicted to increase as age increases, with higher body mass index. Participants with a BMI > 30 have a higher predicted probability of developing hypertension than individuals with a BMI< 30 (Figure 1b).

## Discussion

Screening campaigns for arterial hypertension are essential for early detection and prevention of cardiovascular disease^20^. These initiatives identify high-risk populations, assess prevention and treatment strategies, and enable timely interventions ranging from lifestyle modifications to pharmacological therapy.

Additionally, such campaigns provide valuable epidemiological data on hypertension prevalence and trends in Morocco. Our study enrolled a large volunteer sample with age and gender distributions consistent with High Commission for Planning (HCP) data ^17^, rendering it representative of the general Moroccan population.

The primary conclusion of our study indicates that the overall prevalence of hypertension in the Moroccan population is 36.9% based on the 2024 ESC Guidelines for arterial hypertension. This rate is higher yet consistent with the first Moroccan national survey conducted in 2000, which reported a prevalence of 33.6%. In this latter BP measurements were performed using Vaquez sphygmomanometer device, while in our study OMRON-automated devices were used^21^.

The apparent increase in hypertension prevalence in Morocco over 24 years can be attributed to demographic factors; the Moroccan population is gradually aging due to higher life expectancy compared to 24 years ago. Rapid urbanization came with different lifestyle and behavioral changes, including changes in dietary habits, physical activity, obesity and overweight. This increase can also be attributed to expanded healthcare access due to the improvement of healthcare infrastructure.

Within the regional context, this high prevalence of arterial hypertension aligns with findings from studies in developing countries with similar sociodemographic characteristics. For instance, the 2009 multicenter epidemiological study on hypertension in the ’Maghreb’ reported an overall prevalence of 45%^22^. Within these countries, Algeria had the highest prevalence (49.5%)^23^, followed by Tunisia (47.4%)^24^. On a broader African scale, the prevalence of hypertension varies significantly; Kenya and Cameroon reported rates of 26.1% and 20.8%, respectively^25,26^. Worldwide, hypertension prevalence is highly variable.

This study underscored an alarming finding; more than half of the participants (52.9%) were unaware of their hypertension condition. The prevalence of hypertension awareness varies significantly between countries; these disparities are probably due to differences in healthcare access and preventive strategies in each country. Georgia (85.4%)^27^, Taiwan (84.7%)^28^, Poland (83%)^29^, and Argentina (81.1%)^30^, exhibit some of the highest awareness rates. On the opposite side of the Mediterranean, Spain and Italy stand at 77.2% and 62.1% respectively^31,32^. In contrast, awareness levels are considerably lower in several African countries, including Ghana (36.5%)^33^, Cameroon (33.1%)^26^, and Malawi with the lowest rate (17.4%)^34^.

Despite the significant medical advancements and the accessibility of effective antihypertensive medications, a substantial proportion of hypertensive patients continues to fall short of attaining the recommended blood pressure targets^35^. This lack of control remains a considerable barrier to achieving sustainable health development goals^10^. In this study, 52.9% of aware hypertensive participants had uncontrolled blood pressure. Comparatively, a study conducted in Meknes, Morocco reported a higher rate (73%)^10^, while the figure for North Africa overall stands at 64%^22^.

Morocco has made notable strides in expanding healthcare coverage and improving its infrastructure; however, our findings underline the rising burden of arterial hypertension. To control this persistent challenge in Morocco, stakeholders must begin by focusing on primary care improvement, to ensure cost-effective and continuous care for hypertensive patients as well as those at high risk of developing arterial hypertension. On the other hand, physicians’ clinical decisions must be evidence-based decisions relying on the latest international guidelines for the management of arterial hypertension. They also must integrate BP screening into all adults’ consultations, raise awareness on the necessity of adopting a healthy, balanced diet, physical activity, and avoid smoking and alcohol consumption. Additionally, individuals in rural areas and low-income groups must have access to affordable antihypertensive medications and health education.

## Conclusion

The results of this opportunistic, volunteer-based screening campaign to assess the prevalence of arterial hypertension in Morocco demonstrated a high prevalence of hypertension among the Moroccan population. It also pointed to a high burden of undiagnosed and uncontrolled hypertension, even among those under pharmacological treatment. Such findings might suggest a poor and ineffective management of hypertension in Morocco. From this perspective, strategies to raise awareness and control of hypertension should be strengthened, not only by focusing on the health system, but rather more via multi-sectoral strategies and approaches that aim to engage local communities such as schools, universities, and other civil associations to increase awareness, and more effective blood pressure treatment to manage the burden of arterial hypertension in Morocco.

## Strengths and limitations

This study had several limitations inherent to its observational design. Despite extensive training of all volunteers in accurate blood pressure monitoring, measurement inaccuracies occurred, necessitating exclusion of some values. Additionally, BP readings may have been influenced by environmental factors such as noise at screening sites. To ensure adequate inter-observer reliability, investigators underwent advanced training in BP measurement to guarantee protocol standardization using consistent procedures and equipment.

Convenience sampling represented another limitation, as only individuals with ready access to public venues where screening occurred were enrolled. Geographic distribution and socioeconomic factors were not assessed, though Morocco’s significant socio-spatial transformation has enhanced mobility and increased rural–urban interaction, facilitating access to urban public spaces for rural populations and vice versa. Future investigations should address these issues with particular attention to demographic characteristics, including educational attainment, socioeconomic status, and geographic distribution. Nevertheless, these limitations are common to epidemiological studies of this nature and are unlikely to substantially impact the overall hypertension prevalence estimates, particularly given our large sample size.

## Acknowledgements

We would like to thank all investigators and Master students of Human Physiology and Pathophysiology of the Faculty of Medicine and Pharmacy of Tangier, and all the participants of this study.

## Funding

The authors report no involvement in the research by any sponsor that could have influenced the outcome of this work.

## Disclosure of interest

The authors certify that there is no conflict of interest with any financial organization regarding the material discussed in the manuscript.

## Data availability statement

The data of the current study are available from the principal investigator.

## Authors’ contribution

1. **Conceptualization:** El hangouche Abdelkader Jalil, Taiek Nora
2. **Data curation:** El hangouche Abdelkader Jalil
3. **Investigation:** Taiek Nora, Belkacem Abderrahmane
4. **Methodology:** El hangouche Abdelkader Jalil, Taiek Nora
5. **Data analysis:** Taiek Nora, Belkacem Abderrahmane
6. **Supervision:** El hangouche Abdelkader Jalil
7. **Validation**: El hangouche Abdelkader Jalil
8. **Writing–original draft:** Taiek Nora, Belkacem Abderrahmane, Aziouaz Faiza
9. **Writing-review and editing:** El hangouche Abdelkader Jalil
